# Machine Learning Predictions and Identifying Key Predictors for Safer Intubation: A Study on Video Laryngoscopy Views

**DOI:** 10.3390/jpm14090902

**Published:** 2024-08-25

**Authors:** Jong-Ho Kim, Sung-Woo Han, Sung-Mi Hwang, Jae-Jun Lee, Young-Suk Kwon

**Affiliations:** 1 Department of Anesthesiology and Pain Medicine, Chuncheon Sacred Heart Hospital, Hallym University College of Medicine, Chuncheon 24253, Republic of Korea; poik99@hallym.or.kr (J.-H.K.); h70sm@hallym.or.kr (S.-M.H.); 2 Institute of New Frontier Research, Hallym University College of Medicine, Chuncheon 24253, Republic of Korea; hsw4070@hallym.ac.kr

**Keywords:** video laryngoscope, machine learning, percentage of glottic opening score, SHapley Additive exPlanations value

## Abstract

This study develops a predictive model for video laryngoscopic views using advanced machine learning techniques, aiming to enhance airway management’s efficiency and safety. A total of 212 participants were involved, with 169 in the training set and 43 in the test set. We assessed outcomes using the percentage of glottic opening (POGO) score and considered factors like the modified Mallampati classification, thyromental height and distance, sternomental distance, mouth opening distance, and neck circumference. A range of machine learning algorithms was employed for data analysis, including Random Forest, Light Gradient Boosting Machine, K-Nearest Neighbors, Support Vector Regression, Ridge Regression, and Lasso Regression. The models’ performance was evaluated on the test set, with Root Mean Squared Error values ranging from 20.4 to 21.9. SHapley Additive exPlanations value analysis revealed that age is a consistent and significant predictor of POGO score across all models, highlighting its critical role in the predictive accuracy of these techniques.

## 1. Introduction

Endotracheal intubation is an essential procedure that ensures safe ventilation during emergency and surgical operations [1]. Video laryngoscopy is the preferred method owing to its high success rate and low complication rate, which varies in effectiveness based on the patient’s physical measurements, directly influencing the difficulty of the intubation process [2,3,4,5].

Existing studies have highlighted that the anatomical characteristics of the neck, patient obesity, and size of mouth opening significantly affect the laryngoscopic view [6,7]. Furthermore, factors such as Mallampati class, sex, and patient age are critical for determining the quality of the laryngeal view [8]. Despite valuable insights from the existing research, the primary focus has been on evaluating the success rates of video-based endotracheal intubation. Although these studies provided important information, they often failed to offer a comprehensive and quantitative analysis of the impact of specific patient characteristics on these outcomes. Most of these studies addressed individual anatomical features, such as neck circumference, jaw flexibility, or visibility of the larynx in isolation. However, the interplay between these characteristics, that is, how they combine and interact to influence the ease or difficulty of intubation, has not been sufficiently explored. The lack of an integrated analysis prevents a holistic understanding of the factors that can predict or enhance the success of intubation procedures. Consequently, anatomical variances are often viewed in silos without a clear methodology for assessing their collective impact on procedural efficacy and safety. This creates a critical research gap, as understanding these interactions could lead to the improved training of medical personnel, better patient outcomes, and the development of more effective tools and techniques for airway management.

This study aimed to develop a predictive model for video laryngoscopic views using advanced artificial intelligence and machine learning techniques. We employed various predictors, including patient age, height, body weight, neck circumference, body mass index (BMI), thyromental distance, sternomental distance, thyromental height, maximum mouth opening distance, Mallampati class, and sex. Analysis of the influence of these variables on video laryngoscopic views is intended to enhance the accuracy of preintubation airway assessments, thereby facilitating more informed clinical decision-making and reducing the risk of complications. This study utilized artificial intelligence and machine learning technologies to comprehensively analyze various variables, such as patient body shape, neck circumference, and mouth size, to predict the likelihood of success in video-endotracheal intubation in ways that were previously impossible.

Moreover, this study leveraged the advancement of machine learning technologies to effectively explore complex data patterns in the medical field. The application of interpretability techniques, such as SHapley Additive exPlanations (SHAP) values, provides detailed information on the factors that most influence model predictions [9], thereby enhancing transparency in the medical decision-making process. This will contribute to the realization of precision medicine in medical settings.

This study conducted a secondary analysis using existing data, which was cost-effective, and explored generalizability through extensive patient data. Our findings will lay a solid scientific foundation for a broad range of medical applications. Secondary analysis employed machine learning to identify and quantify the physical and physiological characteristics of patients that affect video laryngoscopic views and their impact using SHAP values. This approach aimed to not only improve the efficiency and safety of airway management but also to establish a methodological foundation for the application of machine learning technologies in clinical settings. This enables a more precise pre-assessment in medical environments, thereby supporting decision-making in endotracheal intubation scenarios.

## 2. Materials and Methods

### 2.1. Study Design

In this study, we employed a secondary analysis approach using machine learning to investigate the factors influencing video laryngoscopy views and the extent of their impact. This secondary analysis is based on data from a previously conducted study by Kim J.H. et al [10]. We utilized data previously collected in the original study along with additional data used for validation [10]. Our goal was to identify and predict the determinants of video laryngoscopic views among existing airway management factors using machine learning.

A previous study conducted at the Chuncheon Sacred Heart Hospital in South Korea targeted patients scheduled for surgery under general anesthesia. This medical institution is affiliated with Hallym University and is located in the northern Yeongseo region of the Gangwon Special Self-Governing Province. It is one of the three Regional Emergency Medical Centers in Gangwon Special Self-Governing Province and serves a vital role in providing emergency medical services to the area. As a comprehensive medical facility, the Chuncheon Sacred Heart Hospital offers a wide range of medical services to the local community and acts as a key provider of surgical care. Hospitals include various specialties and support diverse patient demographics. A previous study from which data for this secondary analysis were drawn included adult patients aged 18 years and older who were scheduled for elective surgery under general anesthesia at a height of 170 cm or more [10]. This patient cohort was selected between 1 November 2021, and 31 March 2023, at the Chuncheon Sacred Heart Hospital, South Korea. The exclusion criteria were patients who had undergone or were scheduled to undergo airway-related surgery, those with intubated airways, those without prior consent to the study, those with unstable cervical spines, those requiring emergency sequential intubation, and those for whom video laryngoscopy was contraindicated. Decisions on patient exclusion were made by an anesthesiologist, independent of the study’s interventions and recordings. All endotracheal intubation data obtained through video laryngoscopy examinations were obtained by a single experienced anesthesiologist with over seven years of expertise in the field of video laryngoscopy. This research adhered to the consolidated reporting guidelines for prognostic and diagnostic machine learning modeling studies (refer to the Table S1). Details of the study design, randomization, and blinding procedures can be found in the original publication [10].

### 2.2. Ethical Approval/Informed Consent

This study was approved by the Institutional Review Board of the Chuncheon Sacred Heart Hospital (IRB No. 2024-03-007). All research strictly adhered to the ethical principles outlined in the Declaration of Helsinki. Vulnerable subjects were excluded from this study, and the requirement for written consent was waived.

In our study, we decided not to specify a predetermined sample size because we believe that including as many participants as possible enhances the generalizability and statistical power of the machine learning outcomes, ensuring robust results. This study was retrospective in nature, and approval was obtained from the Institutional Review Board to ensure ethical conduct and participant safety. Therefore, we aimed to maximize participant involvement to enhance the validity and reliability of the research findings, and we utilized all the data collected in a previous study.

### 2.3. Definition of Airway-Associated Factors and Data Collection

**Figure 1:**
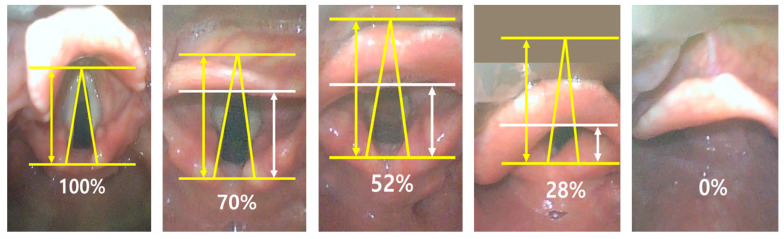
Examples of Video Laryngoscopic Views Corresponding to Various POGO Scores.

The video laryngoscope used in this study was an AceScope (Acemedical, Seoul, Republic of Korea) (Appendix A). The specifications for the AceScope video laryngoscope indicate a resolution of 640 × 480 pixels at a maximum frame rate of 30 frames per second, an angle of view/depth of field of ~71.9 degrees diagonally, with an allowable range of variation of approximately ±15%, and a light source of camera LED providing over 150 lx Illumination. The percentage of glottic opening (POGO) was used as a metric for outcome assessment. The POGO score denotes the percentage of glottic visibility during laryngoscopy, spanning from the interarytenoid notch to the anterior commissure [11]. To ensure a consistent and accurate evaluation of the POGO scores, we recorded videos of the intubation process for all participants. POGO scores were assessed from these recordings, specifically using still images captured at the point of maximum vocal cord exposure. To enhance the reliability, the intubator did not take part in the assessment of POGO scores. The POGO score assessments were reviewed and confirmed by two experienced investigators. In case of the glottic opening being invisible, a POGO score of 0 was assigned. All POGO scores were measured using a video laryngoscope under general anesthesia via the oral route. Each patient underwent anesthesia induction with propofol and rocuronium, and intubation was performed under neuromuscular monitoring when the train-of-four value reached 0. To ensure consistency across all procedures, the depth of blade insertion was strictly controlled. Our standardized approach aimed to insert the blade tip precisely into the patient’s vallecula regardless of the specific blade size used. While auxiliary techniques such as Backward, Upward, and Rightward Pressure (BURP) or lifting the epiglottis are commonly employed in clinical practice when low POGO scores are observed, the dataset in this study does not include POGO scores obtained after applying BURP or any other auxiliary techniques. Figure 1 shows an example of a video laryngoscopic view corresponding to the POGO score.

Factors associated with the airway included the modified Mallampati classification, thyromental height, thyromental distance, sternomental distance, distance from the mouth open, and neck circumference.

The modified Mallampati classification evaluates the space available for manipulation by visually assessing the distance from the base of the tongue to the mouth, providing a visual assessment [12]. It serves as an indirect indicator of intubation difficulty and is categorized as follows.

Class I: Soft palate, uvula, fauces, and pillars visible.
Class II: Soft palate, major part of uvula, and fauces visible.
Class III: Soft palate and base of uvula visible.
Class IV: Only the hard palate visible.

The thyromental height was defined as the distance between the anterior border of the thyroid cartilage and the mentum notch [13]. The thyromental distance was measured along a straight line from the prominence of the thyroid cartilage to the lower border of the mandibular mentum with the head fully extended [14]. The sternomental distance was assessed as the straight distance between the upper border of the manubrium sterni and the bony point of the mentum, with the head fully extended and the mouth closed [15]. Neck circumference was measured at the mid-neck, between the mid-cervical spine and mid-anterior neck, with subjects standing upright and facing forward while ensuring relaxed shoulders [16].

Except for thyromental height, these parameters were measured in patients in the sitting position, whereas thyromental height was measured in patients in the supine position. All the measurements were performed by a single research nurse.

The data for this analysis were extracted from the original database. The original methodology for data collection, including the measurement of POGO scores, is described in detail in the original publication [10].

### 2.4. Factors Considered and Addressed in This Study

Known predictors and confounders of video laryngoscopy include various factors that can influence the extent of the laryngeal view obtained through video laryngoscopy. The predictors, confounders, and methods used in this study are described below.

- Patient Anatomy: Anatomical variations, such as neck circumference, mouth opening, thyromental height, thyromental distance, and sternomental distance, and the presence of abnormalities can significantly affect laryngeal visualization. To address this issue, we included airway-related anatomical characteristics as predictors in our analysis. Patients with airway-related anatomical abnormalities were excluded.
- Operator Experience and Skill: Operator proficiency is crucial to achieving optimal visibility during video laryngoscopy. To enhance the reliability, the intubator did not take part in the assessment of POGO scores.
- Presence of Blood, Secretions, or Debris: Blood, secretions, or debris can obstruct the camera lens and impair visualization. To mitigate this, patients with potential obstructive factors were excluded, intubation was performed under optimal conditions, and an optimal laryngoscopic view was preserved.
- Use of Stylets: Assistive devices, such as stylets, may affect tube insertion ease and laryngeal visibility. For standardization, a stylet was used in all cases to maintain an optimal intubation environment.
- Hemodynamic Status: Hemodynamic stability is crucial for airway patency and visibility. Therefore, only electively stable surgical patients were included in this study.
- Patient Position During Intubation: Patient positioning affects airway alignment and visualization. Hence, for consistency, all patients were placed in a supine position with the same pillow configuration.
- Use of Sedatives or Muscle Relaxants: Sedatives and muscle relaxants can alter airway dynamics and affect visualization. Therefore, sedatives were withheld before anesthesia, and only rocuronium was used as a muscle relaxant. Intubation was performed when neuromuscular monitoring indicated complete muscle relaxation with a train-of-four count of 0.

By addressing these factors, our study aimed to minimize confounding influences and provide reliable information on the factors that affect video laryngoscopic visualization.

### 2.5. Data Preprocessing, Transformation, and Feature Engineering

In terms of the data preprocessing strategy, we aimed to exclude missing and outliers. We did not perform a dimensionality reduction on the data to assess the effects of known airway-related features on POGO. In addition, we did not normalize or standardize the numerical values of the individual features to ascertain their respective effects. Transformation of categorical variables like ‘Mallampati class’ into categorical data types for more efficient processing. The rows with missing values were excluded to ensure data quality and consistency. This step was crucial for maintaining statistical validity across model evaluations.

The data were divided into training and test sets to accurately evaluate the performance of the predictive models. Specifically, the dataset was split at an 80:20 ratio, with 80% of the data allocated for training and the remaining 20% used for testing. This splitting strategy is commonly used in machine learning to provide a substantial amount of data for learning while preserving a significant portion of the unbiased evaluation of the model [17]. The stratification based on ‘POGO’ scores ensured that both sets were well-represented across the entire range of scores, which is critical for maintaining the model’s ability to generalize to new, unseen data.

### 2.6. Model Development and Validation

This study utilized a comprehensive array of machine learning algorithms to analyze the data, including Random Forest, Light Gradient Boosting Machine (LightGBM), K-Nearest Neighbors, Support Vector Regression, Ridge Regression, and Lasso Regression. These models were implemented using the scikit-learn library (available at https://scikit-learn.org/stable/, accessed 8 May 2024) and the LightGBM package (available at https://lightgbm.readthedocs.io/en/stable/, accessed 8 May 2024).

Model performance was rigorously evaluated using metrics such as the Root Mean Squared Error (RMSE), Mean Squared Error (MSE), Mean Absolute Error (MAE), and Coefficient of Determination (R^2^ score). To identify the optimal model configurations, comprehensive hyperparameter tuning was conducted using GridSearchCV [18], which explored the predefined parameter spaces for each algorithm. This process was essential to ensure the selection of the most effective model configurations for the predictive tasks.

### 2.7. Augmentation and Robustness Testing

Data augmentation techniques are applied to enhance the robustness and generalizability of the model. Log transformation was utilized for Continuous variables were log-transformed to normalize their distributions. Additionally, random noise was introduced into the transformed data to simulate various operational conditions and test the resilience of the models to perturbations. We increased the training dataset size by 100% by generating synthetic examples, which were created by applying random perturbations to continuous variables.

### 2.8. Interpretability with SHAP Values

To enhance the understanding of the factors influencing model predictions, SHAP values were computed. This method quantitatively assesses the contribution of each feature to the prediction outcomes, thereby providing deeper insights into the decision-making processes of the models. This analytical approach is crucial for identifying key predictors affecting the video laryngoscopy view, thereby aiding clinicians in enhancing the safety and efficiency of intubation procedures. The SHAP values were calculated using Python’s SHAP library (available at https://shap.readthedocs.io/en/latest/, accessed on 8 May 2024). This systematic methodology underpins the goal of delivering a comprehensive analysis of the influential factors in video laryngoscopy.

For all computational analyses, the Google Colab platform (Google LLC, Mountain View, CA, USA) was employed, leveraging its cloud-based Python environment (Python version 3.7) for enhanced processing capabilities. Descriptive statistics for airway-related factors were presented differently based on the nature of the variables. Continuous variables were summarized using means and standard deviations, while categorical variables were described by frequencies and percentages. We also performed the Mann–Whitney U test to evaluate the statistical significance of the differences between POGO scores in the training and test datasets.

## 3. Results

This study included 212 participants: 169 assigned to the training set and 43 assigned to the test set. After applying data augmentation techniques, the training set was expanded to include 338 cases, enhancing the robustness and diversity of our dataset for model training.

### 3.1. Training and Testing Datasets

Descriptive statistics for the training and testing datasets are presented in Table 1, detailing the median values and interquartile ranges (IQR) for continuous variables and frequencies with percentages for categorical variables. We calculated the median, 25th, and 75th percentile values to compare the POGO scores between the training and test datasets. For the training dataset, the median POGO score was 91.80, with the 25th percentile at 71.90 and the 75th percentile at 100.00. For the test dataset, the median POGO score was 87.80, with the 25th percentile at 69.95 and the 75th percentile at 100.00. There was no statistically significant difference between the two groups (*p* = 0.741).

### 3.2. Performance of Predicting Models

Appendix B presents the hyperparameters of the best model obtained using GridSearchCV. The performances of the various regression models were evaluated using a test set, and the results are summarized in Table 2. Overall, the Ridge and Lasso Regression models demonstrated the best performance in predicting the outcomes of the test set, indicating their potential suitability for modeling this type of data.

### 3.3. Average Impact Magnitude of Features on Model Output: Comparative Analysis of Mean Absolute SHAP Values across Different Models

**Figure 2:**
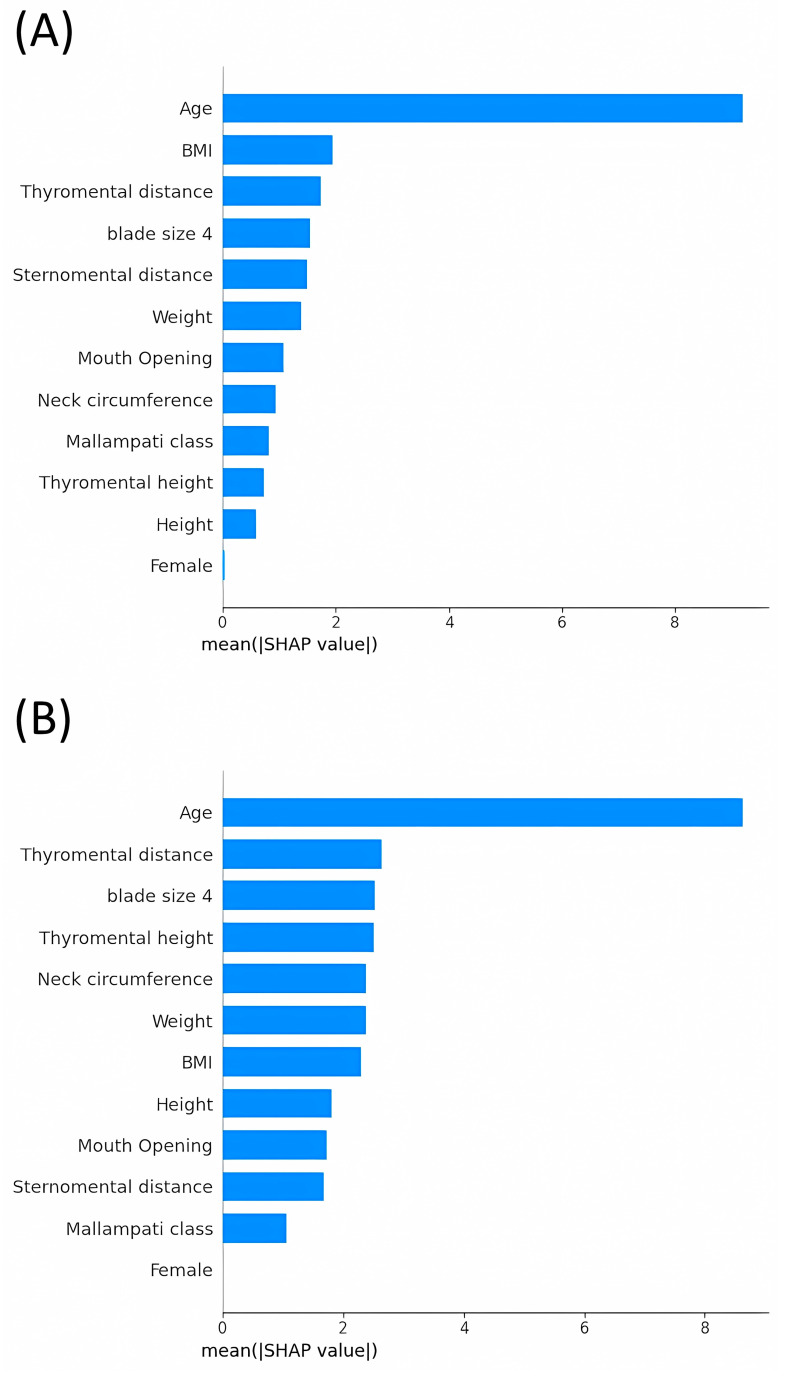

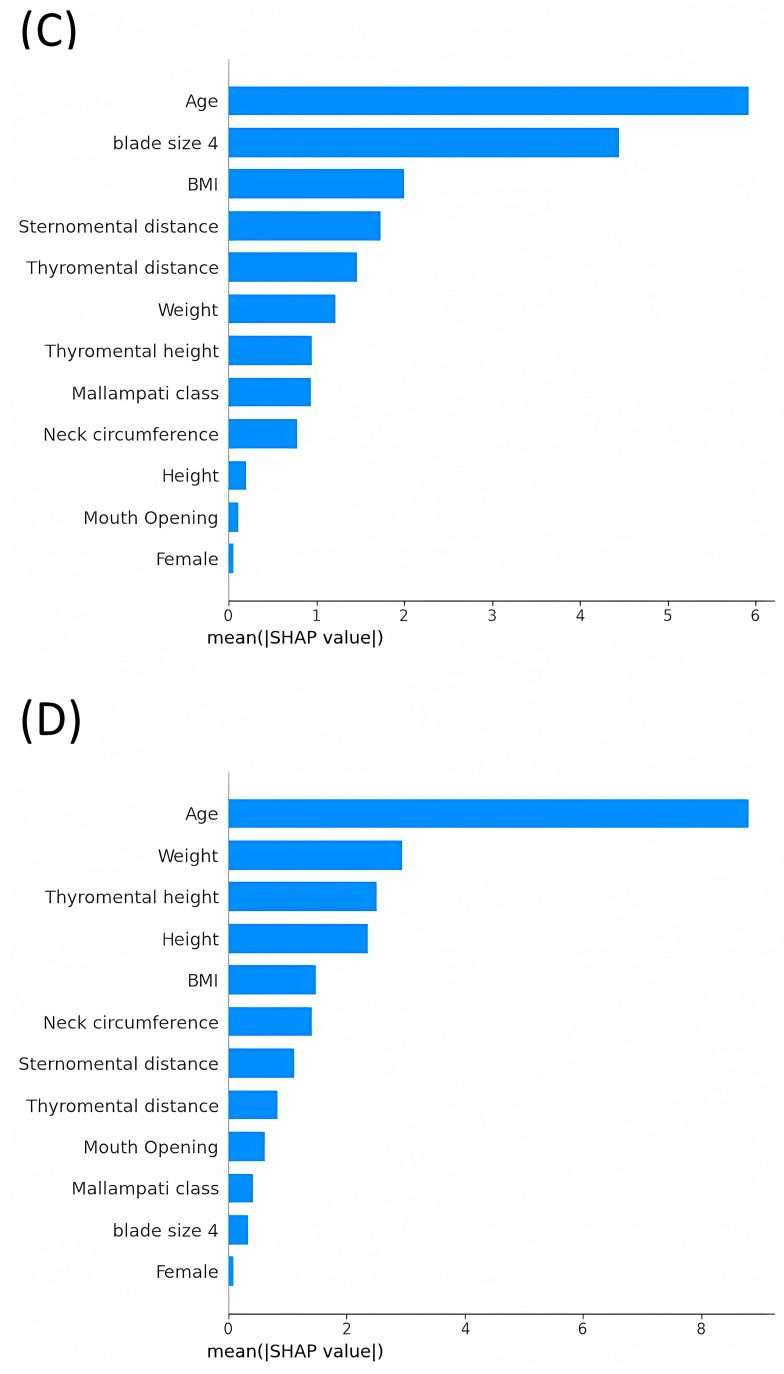

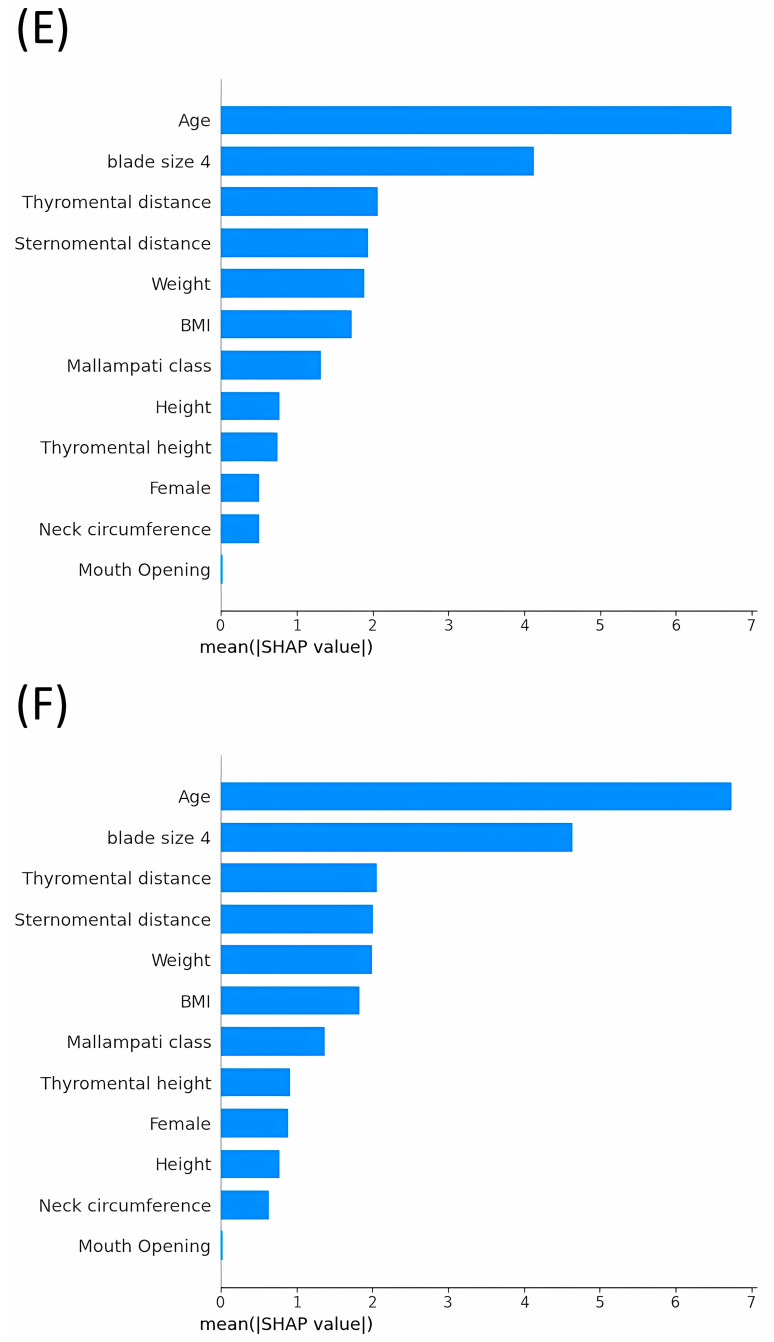
Average Impact Magnitude of Features on Model Output. (**A**), Random forest; (**B**), Light Gradient Boosting Machine; (**C**), Supportive Vector Machine; (**D**), K-Nearest Neighbors; (**E**), Ridge Regression; (**F**), Lasso Regression. BMI, body mass index; SHAP, SHapley Additive exPlanations.

Value analysis of the mean absolute SHAP value provides insights into the influence of individual features across multiple machine learning models used in predicting video laryngoscopic views. We included models such as Random Forest, LGBM, K-Nearest Neighbors, Support Vector Regression, Ridge Regression, and Lasso Regression (Figure 2).

### 3.4. Analysis of SHAP Values: Evaluating the Impact on Model Outputs across Various Machine Learning Models

**Figure 3:**
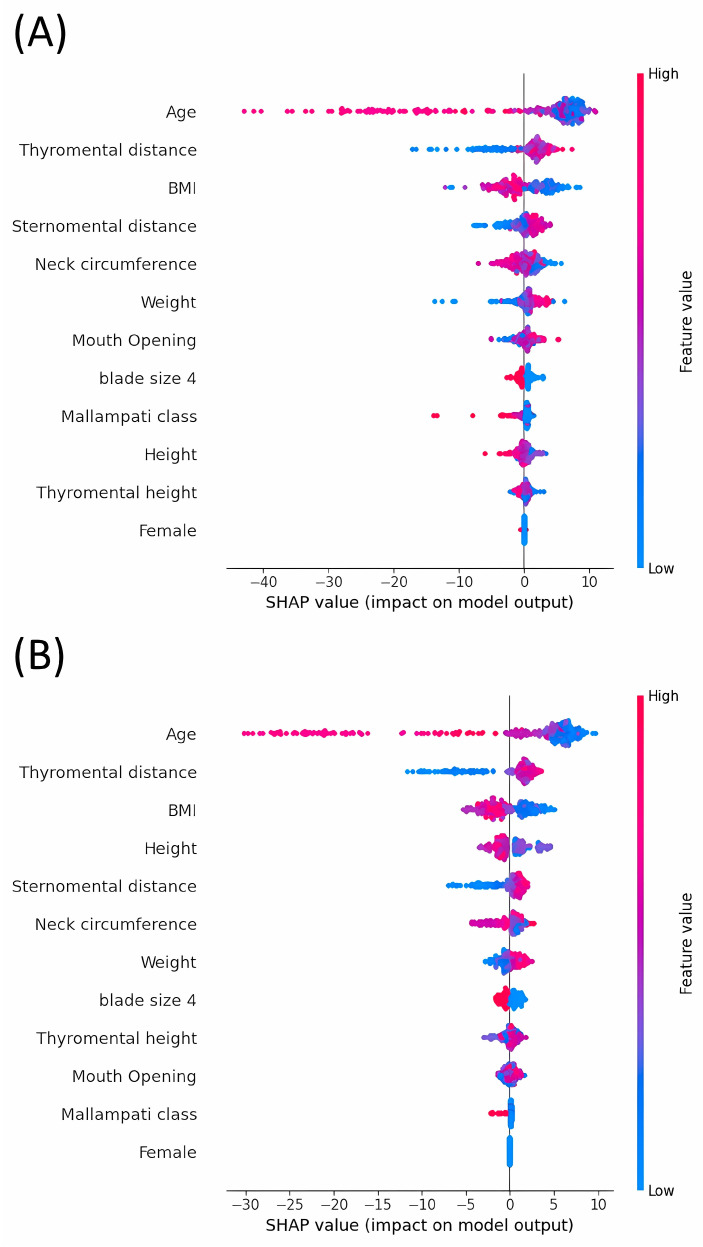

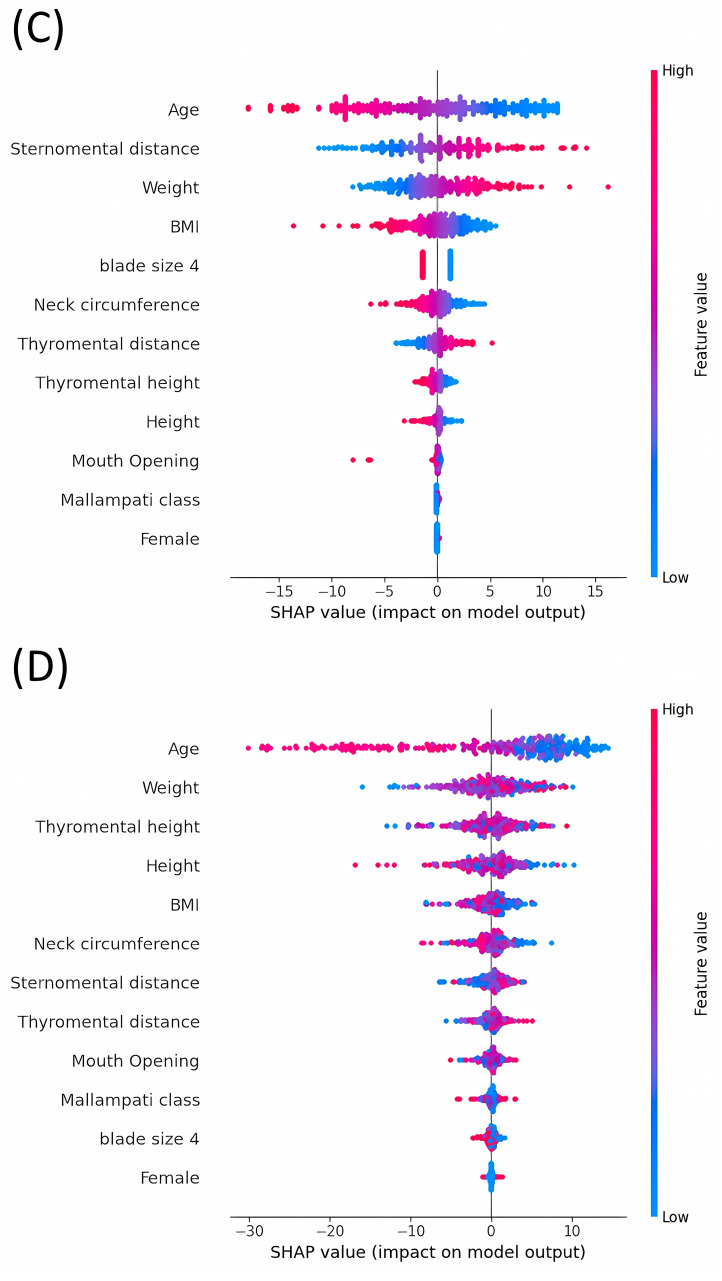

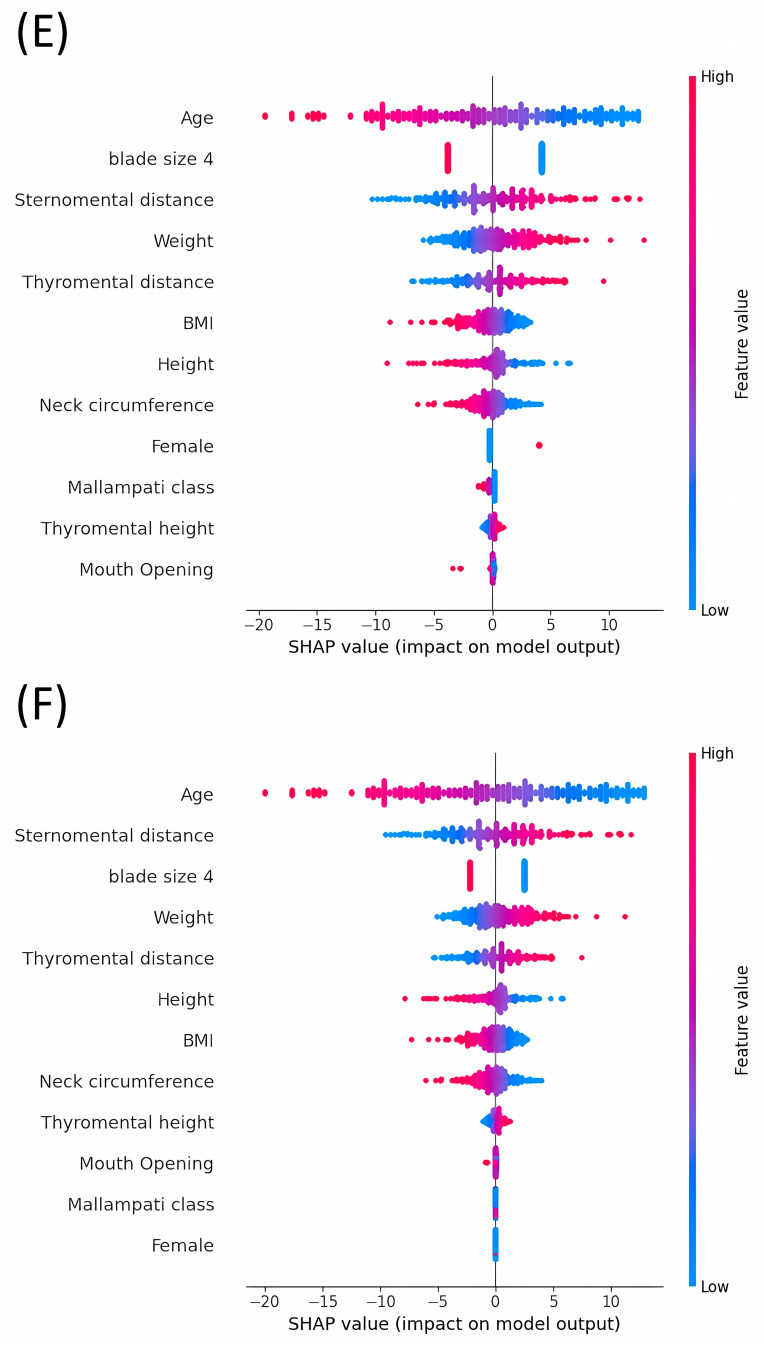
SHapley Additive exPlanations (SHAP) value of features on Model Output. (**A**), Random forest; (**B**), Light Gradient Boosting Machine; (**C**), Supportive Vector Machine; (**D**), K-Nearest Neighbors; (**E**), Ridge Regression; (**F**), Lasso Regression. Age, BMI, neck circumference, mouth opening, thyromental height, thyromental distance, sternomental distance, weight, height, and Mallampati class are coded with increasing values in shades of red and decreasing values in shades of blue. Female is indicated in red, male in blue, blade size 3 in blue, and blade size 4 in red. Positive SHAP values indicate that the presence or higher value of a feature increases the predicted probability of a higher POGO score. BMI, body mass index; POGO, percentage of glottic opening; SHAP, SHapley Additive exPlanations.

Figure 3 provides information on the relative importance and impact of various features used to predict outcomes across six different machine learning models. Across all the models, age consistently emerged as a key predictor, affirming its importance in clinical settings for predicting outcomes.

### 3.5. Univariate Regression Analysis Using Age

We evaluated the predictive power of age alone for the POGO score using a univariate linear regression model. The performance of the model was quantified using several statistical metrics that are essential for understanding how well age predicts the POGO score in isolation from other variables.

- MSE: 503.255
- RMSE: 22.433
- MAE: 16.805
- R^2^: 0.1836

## 4. Discussion

This study initially included 212 participants divided into a training set of 169 individuals and a test set of 43 individuals. To enhance the diversity and robustness of the dataset, data augmentation techniques were applied to expand the training set to 338 cases. The Ridge and Lasso Regression models exhibited the best performance on the test set, as indicated by the lowest RMSE and highest R^2^ values, suggesting their suitability for predicting outcomes using this type of data. MAE values represent the average deviation of the predicted POGO scores from the actual scores. Clinically, an MAE of approximately 15–16 suggests that the predicted scores differ from the actual scores by approximately 15–16 percentage points, on average. Although this indicates a reasonable level of accuracy, it also highlights the need for further refinement and model tuning to achieve higher predictive precision, which can be more impactful in clinical settings. SHAP analyses revealed that age was the most influential predictor across all models. Other significant features varied by model but commonly included weight, thyromental height, and BMI. The Ridge and Lasso models were particularly sensitive to changes in features related to body size, whereas ensemble models such as Random Forest and LGBM emphasized both physical measurements and demographic factors.

The prediction of difficult laryngoscopy using direct laryngoscopy has been extensively studied. Historically, these studies relied on classical statistical methods to identify factors such as limited mouth opening [19], thick neck [20], short thyromental distance [14], thyromental height [21,22], sternomental distance [15], and the absence of teeth [23] as predictors of difficulty. However, recent advancements have shifted the focus towards leveraging artificial intelligence to enhance predictive accuracy [24,25,26,27,28,29]. For instance, Yamanaka S. and colleagues utilized demographics and initial airway evaluations from emergency settings to predict difficult airways, finding that a machine learning model surpassed the performance of the modified LEMON (Look, Evaluate, Mallampati, Obstruction, Neck mobility) criteria in predicting first-pass success [28]. Similarly, Hayasaka T. and colleagues used deep learning to analyze face images classified as ‘easy’ or ‘difficult’ based on the Cormack–Lehane grade by anesthesiologists [29]. Their model, which linked patient facial images with intubation difficulty, successfully identified challenging cases by recognizing facial contours. Moreover, advancements in neural network applications have enhanced the prediction capabilities of such models, surpassing those of conventional methods. Xia et al. reported an artificial intelligence model capable of identifying difficult video laryngoscopies [27]. Their model utilized a neural network that incorporated basic characteristics, medical histories, bedside tests, and seven facial images as predictive variables. They highlighted AI-based facial analysis as a promising technology for predicting the difficulty of video laryngoscopic examinations, and models developed using neural networks demonstrated higher predictive performance than traditional methods. Despite the growing use of video laryngoscopes, there remains a notable gap in research that specifically focuses on the predictors of video laryngoscopic views. To the best of our knowledge, no existing studies have addressed the prediction of the POGO score, a continuous outcome measure, using these advanced technologies. This gap presents a unique opportunity for future research to explore and validate AI-driven models for predicting POGO scores, potentially revolutionizing the approach to anticipate difficult tracheal intubations and enhance clinical outcomes.

Our study expands upon previous attempts to construct multivariate predictors of intubation difficulty, notably those that employed more conventional frequentist statistical methods. Research suggests that combining multiple risk factors for difficult intubations can improve the predictive accuracy of assessments [30,31,32]. However, a large-scale cluster randomized trial by Nørskov AK et al. [33]), which evaluated the effectiveness of the Simplified Airway Risk Index (SARI) in predicting difficult tracheal intubations, contradicts these findings. Unlike our approach, which leverages advanced machine learning techniques, SARI is grounded in traditional risk modeling. Despite the comprehensive nature of their study, involving over 64,000 participants, Nørskov et al. found no evidence that the SARI model reduced unanticipated difficult airways compared to usual airway assessment methods. However, our approach differs significantly from that of Nørskov AK et al. in several key aspects: our study and the study by Nørskov AK et al. fundamentally diverge in terms of their primary outcomes. While our research focuses on predicting difficult laryngoscopy, Nørskov AK et al. targeted unanticipated difficult intubation. Our focus on difficult laryngoscopy aimed to enhance predictive accuracy for one of the earliest challenges in airway management, potentially reducing the incidence of unanticipated difficult intubations as a secondary benefit. We integrated machine learning algorithms capable of detecting complex patterns and interactions among predictive variables, which might be overlooked by traditional models. Although the SARI model utilizes a predefined set of risk factors, our model dynamically evaluates the importance of each predictor using techniques, such as SHAP, enhancing our understanding of how individual factors contribute to the risk of difficult laryngoscopy. Of course, the absence of significant improvements in predicting difficult intubations in the study by Nørskov AK et al. raises important considerations for our work. This underscores the inherent challenges of predicting difficult airways and highlights the potential limitations of relying solely on traditional multivariate models. Our findings suggest that incorporating machine learning could enhance predictive accuracy but also prompt a cautious interpretation of these advancements. Although our model shows promise, the clinical applicability of machine learning models must be validated in real-world settings to ensure that they can reliably inform clinical decision-making without introducing new risks. 

In this study, the POGO score was used to evaluate the outcomes of video laryngoscopy. Video laryngoscopy offers a magnified, high-resolution view of the airway, significantly enhancing its ability to precisely determine the visible portion of the glottic opening [2]. By quantifying this visibility as a percentage, the POGO score aligned well with the detailed visual data provided by video laryngoscopy, enabling more accurate and detailed assessments. The quantitative nature of the POGO score significantly diminishes subjective interpretations and reduces observer variability, which are issues prevalent in the qualitative Cormack–Lehane grading system. This objectivity is especially valuable in video laryngoscopy, in which the clarity of the visual output can be precisely quantified using the POGO scale. Furthermore, in clinical practice, the exact measurements provided by the POGO score assist in making informed decisions regarding the necessity of alternative intubation strategies or devices. In the realm of research, employing POGO scores in video laryngoscopy studies facilitates a more detailed analysis of airway visibility and its influence on intubation challenges, success rates, and potential complications. This detailed approach could aid the development of evidence-based guidelines and protocols, thereby enhancing the overall effectiveness and safety of airway management practices.

In our study, we measured the POGO score during video laryngoscopy. It is important to note that a low Cormack–Lehane score is directly correlated with difficult intubation when using a convectional direct laryngoscope, but successful intubation can occur even when the POGO score is as low as zero [34]. Successful intubation can occur even when the POGO score is as low as zero. Nonetheless, lower POGO scores typically indicate that the additional manipulation of the laryngoscope or adjustment of the patient’s position may be necessary to achieve a better view and facilitate intubation. These manipulations can extend the duration of hypoxia (hypoxic time), particularly if multiple attempts are required to secure airways. Furthermore, increased manipulation can increase the risk of airway trauma [35,36]. Thus, predicting the POGO score is crucial as it aids in anticipating potential challenges in airway management. By effectively predicting lower POGO scores, clinicians can better prepare for difficult intubations, potentially reducing the risks associated with prolonged intubation attempts and minimizing airway trauma. This preparation can improve patient outcomes and safety during anesthesia and critical care procedures.

AI models, particularly machine learning and deep learning models, operate based on complex algorithms; therefore, it can be difficult to understand the process of deriving the results [37]. This implies that it is difficult to determine exactly which factors influence the results of the model. Visualization techniques, such as Gradient-weighted Class Activation Mapping, can help image analysis models show which areas of the image influence the results but are limited in their ability to quantify their impact [38]. However, the SHAP value is of great help in interpreting AI results by quantitatively presenting the influence of each feature on the model prediction. The Shapley contribution is a method derived from game theory that calculates the contribution of each feature to the model prediction [39]. The SHAP value numerically represents the influence of each feature on the model prediction. This provides a clear picture of the features that have the greatest impact on the prediction results and whether each feature has a positive or negative impact [9]. The SHAP value helps to understand the inner workings of a model by calculating the influence of each feature on the prediction result. This allows us to understand which features the model considers important and what combination of features it uses to produce the prediction results.

The trend observed in our study, where older age tended to lower the POGO score and younger age tended to increase it, could be significant. Additionally, the fact that the performance of the univariate linear regression model, which included only age, was not significantly inferior to that of the multivariate model suggests that the effect of age on the POGO score is substantial. These observations can be explained by several physiological and anatomical factors that vary with age. Older adults often experience a reduction in tissue elasticity, including the tissues of the neck and airway [40]. This can make the airway more rigid and less adaptable to the manipulations required during laryngoscopy, potentially obstructing the view of the glottis. Degenerative changes in the cervical spine such as osteophytes (bone spurs) and reduced intervertebral disc height limit neck mobility [41]. This decreased mobility makes it difficult to achieve the optimal positioning for laryngoscopy, thereby reducing the POGO score. Older adults are more likely to have missing teeth or use dentures, which can alter the anatomy of the mouth and affect the entry and maneuverability [23]. Regarding increased comorbidities, conditions such as arthritis, obesity, and diabetes are more prevalent in older populations. These conditions can complicate airway management because of factors such as increased neck circumference (obesity) and joint stiffness (arthritis) [16,42]. In contrast, younger patients typically have more elastic tissues and greater flexibility of the cervical spine, facilitating easier and more effective manipulation during laryngoscopy, which helps achieve a higher POGO score. These age-related factors can contribute to the variance in POGO scores, with older patients generally presenting more challenges in achieving a clear view during laryngoscopy. Although our hypothesis was not initially centered on age as a significant factor, our findings indicate that age is a substantial predictor of POGO scores. We acknowledge that our study’s findings are not definitive and that age-related effects on POGO scores should be considered as a hypothesis that requires further investigation. This underscores the importance of additional research exploring the influence of age on airway management.

In our dataset, 21 patients had POGO scores of less than 50. Notably, one patient had an initial POGO score of 0, which marginally increased to 26.4 after the epiglottis was lifted. Despite employing the BURP maneuver, the POGO score did not improve, leading to concerns regarding excessive applied pressure. This necessitated a change in the blade size and a second intubation attempt. Although the number of such cases was limited and not statistically significant for broad generalization, these observations highlight important considerations. When models predict low POGO scores, they may indicate scenarios in which additional maneuvers do not sufficiently enhance the intubation view. Consequently, practitioners should be aware that even with intervention, certain cases may still present significant challenges, potentially necessitating alternative techniques or equipment adjustments to achieve successful intubation.

Although traditional statistical tests calculate the power based on the ability to detect a predefined effect size with a certain degree of confidence, the concept of power in machine learning can be interpreted through the lens of model performance and generalizability. In the context of our grid search, the extensive use of cross-validation acts as a surrogate measure of a model’s power by demonstrating its ability to consistently perform well across different data segments. This robust validation process leads to the selection of model configurations that are not only tailored to fit the training data but are also capable of achieving reliable and generalizable results across unseen datasets. Additionally, because ensuring the selection of the most effective model configurations was crucial for our predictive tasks, this was achieved by evaluating the model performance metric, RMSE, across the cross-validation folds, which further supports the reliability of our model’s predictive power. The comprehensive evaluation process provided by GridSearchCV is pivotal for minimizing the potential for model overfitting and optimizing the general applicability of the model in real-world settings.

This study, which investigated age and other predictors of POGO scores on video laryngoscopy, has some limitations that could affect the interpretation and applicability of the findings.

- Sample Diversity: The study was limited by the relatively small number of participants, which may restrict the generalizability of the findings. Additionally, the sample lacked diversity in terms of ethnicity, sex, and health status, which potentially limits the applicability of the results to a broader population. Additionally, despite the implementation of data augmentation techniques, our models exhibited challenges in accurately predicting lower POGO scores. This limitation primarily stems from the under-representation of cases with low POGO values in both the training and test datasets. Video laryngoscopy inherently yielded lower POGO scores than direct laryngoscopy. This discrepancy likely contributes to the models’ decreased ability to effectively predict such outcomes, thus affecting the overall performance of our predictive models. Future studies should focus on enhancing data collection strategies to better represent lower POGO score scenarios, potentially improving model accuracy and generalizability across various clinical conditions.
- Technological Variations: Variability in the equipment used, such as different types of laryngoscopes and variations in video quality, could have affected the visibility of the glottic opening. These variations may have influenced the accuracy of the POGO score assessments.
- Generalizability of the Findings: The findings were derived from specific clinical settings, including emergency rooms, emergency surgeries, and intensive care units. Consequently, these results may not be directly applicable to other clinical environments or routine surgical settings. Additionally, it is important to note that the POGO scores included in our study were recorded prior to performing any maneuvers, such as BURP or lifting the epiglottis. These maneuvers can significantly alter the POGO scores, and their exclusion ensures consistency in our data but may limit the applicability of the findings to scenarios where such techniques are employed. All patients were positioned supine using a standardized pillow designed to provide consistent support and alignment. However, this standardized pillow does not guarantee an exact angle of head positioning. This is critical for reproducibility and understanding of the influence of age on POGO scores. Limited head mobility in older patients may contribute to lower POGO scores, highlighting the importance of standardized head positioning in future studies.
- Temporal Factors: The relevance of the study may diminish over time owing to technological advancements and changes in clinical practice. Such changes could alter the techniques used in video laryngoscopy, thereby affecting the validity of the conclusions of future studies.
- Limitations of POGO Score Selection: We used the POGO score as the primary outcome measure to assess airway visibility during intubation. Although the POGO score offers detailed visibility metrics and quantifies the visual access to the glottis, it has not been widely adopted in clinical practice and may not fully address the clinical nuances associated with airway management. Specifically, the POGO score, by focusing solely on the percentage of glottic opening visible, might not adequately distinguish between the varying degrees of difficulty encountered in challenging intubations typically classified as Cormack–Lehane grades 3 or 4. Critically, as noted by the reviewer and evident in the practices of many airway experts, a POGO score greater than 0 generally indicates a relatively easier airway compared to a score of 0, often corresponding to Cormack–Lehane grades 1, 2a, or 2b. However, this classification highlights a potential limitation in the ability of the POGO score to provide actionable clinical insights for more challenging scenarios, where detailed differentiation between more nuanced grades of visibility could inform better management decisions. To address these concerns, further research is needed to evaluate the effectiveness of POGO scores in predicting truly difficult intubations and explore how they might be integrated or adapted for broader clinical use.
- Weak Performance: Our model exhibits weak performance, which likely indicates the presence of other factors or variables not included in the model that significantly influence the outcomes. In practice, the low explanatory power of the model limits its utility in clinical applications. Collaboration with clinical practitioners is essential for validating and refining a model based on real-world data and feedback. Continuous research on better predictive models is necessary to enhance predictive accuracy and clinical relevance. Despite its limitations, our model can still provide valuable insights into the factors affecting difficult intubation. It can serve as an educational tool in training scenarios to highlight potential issues and considerations in airway management, emphasizing the importance of preparing for various challenges that may arise during intubation.

Despite these limitations, our findings have important clinical implications. The ability to predict POGO scores using machine learning models based on pre-intubation assessments offers a valuable tool for preoperative planning and risk stratification by anesthesiologists and clinicians. Although the models currently show a range of performance metrics with further refinement and validation, they hold promise for enhancing clinical decision-making and improving patient outcomes. Future studies should aim to integrate additional clinical variables and optimize model performance, thereby enhancing the practical utility and reliability of these predictive tools in various clinical contexts.

## 5. Conclusions

The application of machine learning to predict video laryngoscopic views holds considerable promise for improving both the safety and efficiency of airway management and potentially enhancing patient outcomes and healthcare operations. The insights gained from our study substantially support preoperative planning and risk assessment phases, enabling medical professionals to better anticipate the challenges associated with intubation procedures. By identifying the key features that most significantly affect the laryngoscopic view, clinicians can effectively prepare for and mitigate potential complications. Future research should consider integrating these predictive models into clinical decision-support systems to offer real-time assistance during emergency and surgical interventions. Further model refinement by incorporating larger and more diverse datasets is recommended to enhance the predictive accuracy and generalizability of these tools.

## Figures and Tables

**Table 1:** Training and testing datasets.

Features		Train Set (n = 169)	Test Set (n = 43)
Age (years)		44 (32 to 58)	46 (32 to 60)
Female		8 (4.73)	1 (2.3)
Thyromental height (cm)		5.1 (4.5 to 5.8)	50.0 (4.3 to 5.3)
Thyromental distance (cm)		9.8 (9.0 to 10.5)	9.5 (8.75 to 10.5)
Sternomental distance (cm)		17.0 (15.5 to 18.0)	16.5 (15.4 to 17.8)
Mouth opening (cm)		5.8 (5.2 to 6.2)	5.8 (5.4 to 6.1)
Neck circumference (cm)		40.0 (38.5 to 42.0)	40.0 (38.1 to 41.6)
Height (cm)		174.4 (172.4 to 178.0)	174.0 (172.2 to 176.7)
Weight (kg)		79.4 (72.9 to 90.0)	80.8 (68.5 to 88.8)
Body mass index (kg/m^2^)		25.7 (23.6 to 28.7)	24.9 (22.8 to 28.7)
Mallampati class	Grade 1	102 (60.4)	26 (60.5)
	Grade 2	46 (27.2)	8 (18.6)
	Grade 3	17 (10.1)	7 (16.3)
	Grade 4	4 (2.4)	2 (4.7)
Percentage of glottic open score		91.8 (71.9 to 100.0)	87.8 (69.9 to 100)

Age, thyromental height, thyromental distance, sternomental distance, mouth opening, neck circumference, height, weight, and body mass index are presented as medians with interquartile ranges. In contrast, the variables Female and Mallampati class are reported as counts and percentages.

**Table 2:** Performance assessment of Machine Learning Algorithms for percentage of glottic open score prediction.

Algorithm	RMSE	MSE	MAE	R^2^
Random Forest	21.606	466.82	16.409	0.1215
Light Gradient Boosting Machine	20.985	440.39	15.677	0.1713
K-Nearest Neighbors	21.905	479.82	16.616	0.0971
Support Vector Regression	20.632	425.68	15.25	0.199
Ridge Regression	20.453	418.32	15.57	0.2128
Lasso Regression	20.551	422.34	15.572	0.2052

MAE, Mean Absolute Error; MSE, Mean Squared Error; R^2^, coefficient of determination; RMSE, Root Mean Squared Error.

**Figure A1:**
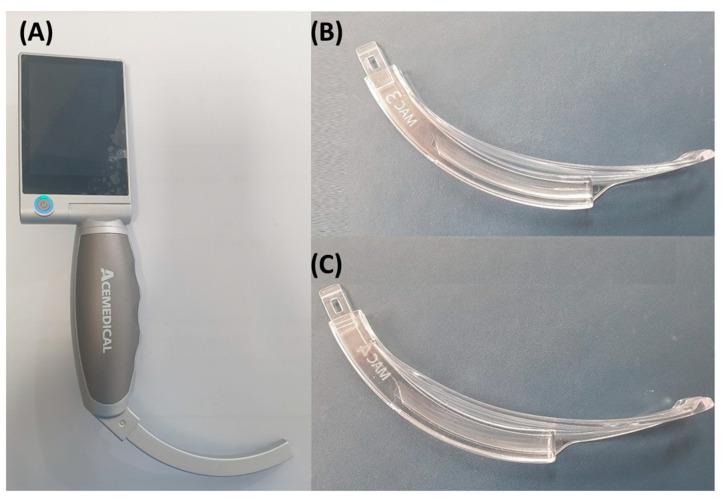
Video laryngoscopy and blade. (**A**) Video laryngoscope; (**B**) curved video laryngoscope blade 3; and (**C**) curved video laryngoscope blade 4.

## Data Availability

Dataset available on request from the authors: The raw data supporting the conclusions of this article will be made available by the authors on request.

## Supplementary Materials

The following supporting information can be downloaded at: https://www.mdpi.com/article/10.3390/jpm14090902/s1.

## Appendix B

Best hyper parameters after GridsearchCV

Best hyperparameters for RandomForest: {‘max_depth’: 20, ‘n_estimators’: 200}
Best hyperparameters for LGBM: {‘learning_rate’: 0.1, ‘max_depth’: −1, ‘n_estimators’: 100}
Best hyperparameters for KNN: {‘n_neighbors’: 7, ‘weights’: ‘distance’}
Best hyperparameters for SVR: {‘C’: 1, ‘gamma’: ‘scale,’ ‘kernel’: ‘linear’}
Best hyperparameters for Ridge: {‘alpha’: 10}
Best hyperparameters for Lasso: {‘alpha’: 0.01}
