# Supplementary material for: Machine Learning Predictions and Identifying Key Predictors for Safer Intubation: A Study on Video Laryngoscopy Views
Source: J Pers Med. 2024 Aug 25;14(9):902. doi: 10.3390/jpm14090902 (PMC11433239; doi:10.3390/jpm14090902)
Supplement: Supplementary file 1 [file jpm-14-00902-s001.zip › jpm-3116296-supplementary.pdf]

Table S1. Consolidated reporting guidelines for prognostic and diagnostic machine learning modeling studies

Multimedia Appendix 1: Author Checklist

The following is the reporting checklist. A response should indicate whether the particular item is documented in the study. If the response to an item is Y then the location in the article should be provided (e.g., section number), and if the response is N or NA then some reasoning should be provided.

#	Item	Y	N	NA	Location / Reasoning
**Study Details**					
1.1	*The medical/clinical task of interest*	o			1-2
1.2	*The research question*	o			1-2
1.3	*Current medical/clinical practice*	o			1-2
1.4	*The known predictors and confounders to what is being predicted / diagnosed*	o			2
1.5	*The overall study design*	o			2-3
1.6	*The medical institutional setting(s)*	o			2-3
1.7	*The target patient population*	O			3
1.8	*The intended use of the ML model*	o			6
1.9	*Existing model performance benchmarks for this task*			o	
1.10	*Ethical and other regulatory approvals obtained*				3
**The Data**					
2.1	*Inclusion / exclusion criteria for the patient cohort*	o			2-3
2.2	*Methods of data collection*	o			3-4
2.3	*Bias introduced due to the method of data collection used*	o			19-20
2.4	*Data characteristics*	o			7
2.5	*Methods of data transformations and preprocessing applied*	o			5-6
2.6	*Known quality issues with the data*	o			20

2.7	*Sample size calculation*			o	
2.8	*Data Availability*	o			21
**Methodology**					
3.1	*Strategies for handling missing data*	o			5-6
3.2	*Strategies for addressing class imbalance*			o	
3.3	*Strategies for reducing dimensionality of data*			o	
3.4	*Strategies for handling outliers*			o	
3.5	*Strategies for data augmentation*			o	
3.6	*Strategies for model pre-training*		o		The data used in the study were specific to the target task, so no additional pre-training was required.
3.7	*The rationale for selecting the machine learning algorithm*	o			5-6
3.8	*The method of evaluating model performance during training*	o			5-6
3.9	*The method used for hyperparameter tuning*		o		6
3.10	*Model's output adjustments*	o			5-6
**Evaluation**					
4.1	*Performance metrics used to evaluate the model*	o			6
4.2	*The cost or consequence of errors*	o			19-20
4.3	*The results of internal validation*	o			8
4.4	*The final model hyperparameters*	o			22
4.5	*Model evaluation on an external dataset*		o		Due to the limited amount of data, external validation could not be performed.
4.6	*Characteristics relevant for detecting data shift and drift*		o		Could not be

					performed due to limited data
**Explainability and Transparency**					
5.1	*The most important features and how they relate to the outcome(s)*	o			8-15
5.2	*Plausibility of model outputs*	o			16-17
5.3	*Interpretation of model's results by an end-user*	o			17-19
